# Mobile telephone delivered contingency management for encouraging adherence to supervised methadone consumption: feasibility study for an RCT of clinical and cost-effectiveness (TIES)

**DOI:** 10.1186/s40814-020-00761-4

**Published:** 2021-01-07

**Authors:** N. Metrebian, E. Carr, K. Goldsmith, T. Weaver, S. Pilling, J. Shearer, K. Woolston-Thomas, B. Tas, C. Cooper, C. A. Getty, R. van der Waal, M. Kelleher, E. Finch, P. Bijral, D. Taylor, J. Scott, J. Strang

**Affiliations:** 1 King’s College London, Institute of Psychiatry, Psychology and Neuroscience, London, SE5 8AB UK; 2 Department of Mental Health, Social Work and Integrative Medicine, School of Health and Education, Middlesex University, London, NW4 4BT UK; 3 Research Department of Clinical Health and Educational Psychology, University College London, London, WC1 7HB UK; 4 Central Acute and Addictions Directorate, South London and Maudsley NHS Foundation Trust, London, SE5 8RS UK; 5 Change, Grow, Live Charity, Management Offices, M4 1NA, Manchester, UK; 6 University of Bath, BAA2 7AY, Bath, UK

**Keywords:** Mobile telephone, Contingency management, Medication adherence, Opioid agonist treatment

## Abstract

**Background:**

Prescription methadone or buprenorphine enables people with opioid use disorder to stop heroin use safely while avoiding withdrawal. To ensure methadone is taken as prescribed and to prevent diversion onto the illicit market, people starting methadone take their daily dose under a pharmacist’s supervision. Many patients miss their daily methadone dose risking withdrawal, craving for heroin and overdose due to loss of heroin tolerance. Contingency management (CM) can improve medication adherence, but remote delivery using technology may be resource-light and cost-effective. We developed an innovative way to deliver CM by mobile telephone. Software monitors patients’ attendance and supervised methadone consumption through an internet self-login at the pharmacy and sends reinforcing text messages to patients’ mobile telephones. A linked system sends medication adherence reports to prescribers and provides early warning alerts of missed doses. A pre-paid debit card system provides financial incentives.

**Methods:**

A cluster randomised controlled trial design was used to test the feasibility of conducting a future trial of mobile telephone CM to encourage adherence to supervised methadone in community pharmacies. Each cluster (drug service/3 allied pharmacies) was randomly allocated to provide patient’s presenting for a new episode of opiate agonist treatment (OAT) with either (a) mobile telephone text message CM, (b) mobile telephone text message reminders, or (c) no text messages. We assessed acceptability of the interventions, recruitment, and follow-up procedures.

**Results:**

Four drug clinics were approached and three recruited. Thirty-three pharmacists were approached and 9 recruited. Over 3 months, 173 individuals were screened and 10 enrolled. Few patients presented for OAT and high numbers were excluded due to receiving buprenorphine or not attending participating pharmacies. There was 96% consistency in recording medication adherence by self-login vs. pharmacy records. In focus groups, CM participants were positive about using self-login, the text messages, and debit card. Prescribers found weekly reporting, time saving, and allowed closer monitoring of patients. Pharmacists reported that the tablet device was easy to host.

**Conclusion:**

Mobile telephone CM worked well, but a planned future trial will use modified eligibility criteria (existing OAT patients who regularly miss their methadone/buprenorphine doses) and increase the number of participating pharmacies.

**Trial registration:**

The trial is retrospectively registered, ISRCTN 58958179.

**Supplementary Information:**

The online version contains supplementary material available at 10.1186/s40814-020-00761-4.

## Key messages regarding feasibility

*What uncertainties existed regarding the feasibility?*

- The accuracy of patient self-login of their own attendance/consumption compared with pharmacy records.
- The ability to recruit the target population.
- The acceptability of delivering CM by mobile telephone to staff, patients, and pharmacists.
- The choice of primary outcomes for the trial.

*What are the key feasibility findings?*

- There was a high level of agreement between patient self-login of attendance and the pharmacy records.
- That patient self-login, the delivery of CM by mobile telephone, and incentives via pre-paid debit cards were feasible.
- Mobile telephone delivered CM was well-liked by patients and staff and acceptable to pharmacists.
- Staff reported that the automated weekly reporting allowed for close monitoring of patients and saved time not having to call pharmacists.
- Recruitment of the target population was not feasible; fewer individuals than expected presented for a new episode of opioid agonist treatment (OAT), were prescribed methadone, or attended one of the three participating pharmacies.

*What are the implications of the feasibility findings for the design of the main study?*

- The target population should not be restricted to such a discrete patient group but instead be modified to include existing patients of OAT who are regularly missing their doses of methadone and buprenorphine
- The number of pharmacies recruited should be increased to ensure the patients are able to participate in the trial.
- The feasibility of revised recruitment procedures and recruitment from this wider population should be assessed with an internal pilot study.

## Background

The majority of those with an opioid addiction are prescribed opioid agonist treatment (OAT) with methadone or buprenorphine for which there is an extensive evidence base [1, 2]. In 2016/2017, there were an estimated 314,000 opioid users in England and Wales of whom approximately 155,000 were in treatment [3]. However, recovery from opioid use disorder is a long-term process and many heroin users relapse leading to OAT having high attrition rates [4]. Patients in OAT are often not achieving abstinence from heroin or other clinical benefits due to non-adherence to medication. Methadone and buprenorphine need to be taken on a daily basis in order to achieve effective maintenance and enable patients to stop heroin use safely and without experiencing excessive symptoms of withdrawal or craving. The Department of Health [5] recommends methadone consumption is supervised in the early stages of treatment in order to improve adherence, safeguard against overdose, and prevent potential for diversion onto the illicit market. Patients starting methadone usually take a daily dose under a community pharmacist’s supervision. A network of pharmacies dispensing OAT medication exists across England. A substantial number of patients occasionally fail to attend the pharmacy to take their methadone with some patients missing multiple doses [6]. Missed doses are likely to lead to withdrawal symptoms and cravings which may lead to heroin use. For OAT patients, each missed dose is of concern. After 3 days missed doses, there is the risk of loss of tolerance to opioids and risk of overdose when the next dose is taken [5]. Consequently, a pharmacist is normally advised to withhold the next day’s dose if a patient has missed three consecutive days, and consult with the prescriber prior to supply. Given this, an additional concern is the pharmacist’s lack of consistent reporting to the prescriber about patient’s missed doses. Ten per cent of pharmacists in England stated they would “never” or “rarely” report one or two missed doses to prescribers but would “usually” report three missed doses [6].

The National Institute for Health and Care Excellence (NICE) recommended that contingency management (CM) be used in UK drug treatment settings to reduce drug use and encourage medication adherence [7]. CM is a behavioural intervention, based on the principles of operant conditioning, and involves the systematic application of positive reinforcement to promote positive behaviour consistent with treatment goals [8]. CM has a large evidence base for its effectiveness in the treatment of substance misuse [9–11]. A recent systematic review of studies using CM/incentives to reinforce medication adherence, but not for OAT, concluded that CM/incentive-based interventions are promising but understudied [12]. While delivering CM can be time-consuming for healthcare professionals and requires integration into healthcare organisational systems, CM delivered by technology might be a resource-light and cost-effective alternative to encourage medication adherence. A systematic review and a meta-analysis of CM delivered by mobile telephone, while finding no prior studies targeting OAT specifically, found CM to be effective [13].

This study focuses on an innovative intervention we have developed to encourage medication adherence by delivering CM via mobile telephone text messages, with a linked system for monitoring and reporting patients’ attendance to prescribers, including early warning alerts of missed doses [14]. It potentially offers considerable benefit for patients, drug treatment services, the NHS, and community pharmacies in the form of a more easily delivered and less resource-intensive intervention.

Our research aimed to investigate the feasibility of conducting a future RCT of the clinical and cost-effectiveness of CM delivered by mobile telephone to encourage adherence to supervised consumption of methadone at community pharmacies among individuals receiving OAT. We also conducted a process evaluation to investigate the acceptability of trial and intervention procedures and to explore factors influencing the pattern of outcomes which we have reported on here.

## Methods

### Study design

This was a three-arm cluster randomised feasibility trial comparing (1) mobile telephone text message CM (MTCM) or (2) mobile telephone text message reminders (MTR) with (3) no telephone text messages (treatment-as-usual) (TAU). To investigate the feasibility of conducting a future RCT, research objectives investigated (a) the willingness of clusters (drug clinics and allied pharmacies) to be randomised, (b) the numbers of eligible patients, (c) rates of recruitment and recruitment procedures, (d) the acceptability of the study to patients, (e) the accuracy of recording/logging in of attendance at the pharmacy, (f) rates of follow-up at 12 weeks, (g) different options for quantifying the primary outcome measure, (h) characterising aspects of the primary outcome measure needed for a sample size calculation for future trial, and (i) the most appropriate secondary outcome measures to determine patient benefit and cost-effectiveness and the availability and usefulness of existing data sets (including pharmacy dispensing data). In addition, contextual factors and treatment process that may impact on outcome were investigated to inform the design of a definitive trial. More detail is provided in the protocol [15].

### Setting

Three drug services (clusters) were recruited from both National Health Service (NHS) (South London and Maudsley NHS Foundation Trust) and non-NHS (“Change, Grow, Live”, London) services and randomly allocated to one of the three arms. Three allied community pharmacies were recruited for each drug service. This was increased from the original target of two after identifying the large numbers of pharmacies providing supervised oral methadone to patients at the participating drug services. Drug services were eligible if they provided OAT where methadone was dispensed for daily supervision at local community pharmacies. Community pharmacies were eligible if pharmacists were willing and able to provide 6 days supervised consumption of oral methadone, the pharmacy had a consultation room on the premises or a separate designated area on the dispensing counter in which participants could consume their oral methadone under supervision, the pharmacy was currently providing supervised consumption of oral methadone to the patients at the drug clinic, and the pharmacy was willing and able to provide dispensing records for participants over the 12-week intervention period. Within each cluster (drug treatment service), participants received the same treatment allocation to minimise the risk of contamination between intervention and control arms.

### Participants

Participants were recruited by drug service staff from patients starting a new episode of OAT (including those newly presenting to the drug service as well as existing patients at the service who were re-starting OAT after not receiving a prescription for ≥ 4 weeks) who were receiving supervised oral methadone at the community pharmacy 6 days a week. Eligible participants included the following: aged > 18 years, prescribed oral methadone, supervised 6 days a week at a participating community pharmacy, owned a mobile phone, and willing and able to provide informed consent. Exclusion criteria included the following: patients unable to read English and would require the service of an interpreter to understand a brief oral description of the study. These individuals were excluded because the two active interventions (mobile telephone text message CM and mobile telephone text message reminders) required participants to be able to read text messages in English. The trial was reviewed by the London South East Research Ethics Committee and received a favourable opinion on 23 November 2018.

### Sample size

One of the aims of the feasibility trial was to estimate parameters needed for a sample size calculation for a larger confirmatory trial. Therefore, no formal sample size calculations were undertaken but we aimed to recruit 60 consenting patients (20 per drug service).

## Processes, interventions, and comparisons

The three drug services were allocated to the three treatment arms using simple randomisation (a 1:1:1 allocation ratio) with sequences generated using a random number generator. The senior statistician (KG) was blind to treatment allocation, but all other members of the study team, individual participants, and staff at the drug service and pharmacy were unblind.

### Interventions

In all arms, over the 12-week intervention period, OAT was delivered in line with existing service protocols and participants used a self-service internet login at the pharmacy to record their attendance and consumption of methadone. There were two active arms (MTCM and MTR) and one treatment-as-usual arm (TAU). In the two active arms, the mobile telephone-based software monitored patients’ attendance and supervised methadone consumption through the internet self-login at the pharmacy and contacted patients via text message. A linked system monitored and reported patient’s attendance and provided early warning alerts of missed doses to patients’ prescribers. In the MTCM arm, financial incentives were delivered via pre-paid debit cards.

**Table:** 

Mobile telephone text message CM (MTCM)	Each time a participant attended the pharmacy and consumed their supervised oral methadone, they received a text message giving praise and earned a financial reward of 50p. If they attended for 6 days consecutively, they earned a bonus reward of £5. The total possible financial reward was £8/week or £96 over 12 weeks. Patients were paid directly via a Bread4Business debit card. If they failed to attend, participants received a “shaping text message” informing them they can still earn 50p if they attend the pharmacy and take their dose the next day.
Mobile telephone text message reminders (MTR)	Participants were reminded via text message each morning and afternoon (if they failed to attend in the morning) to attend their pharmacy for their supervised medication.
Treatment-as-usual (TAU)	Participants received no text messages.

The text message CM or reminders were paused if a participant failed to attend and consume their dose on three consecutive days, in line with clinical guidance, and were re-started again when the participant was re-instated on their methadone. Prescribers at clinics allocated to MTCM or MTR received weekly reports via email of patient attendance and an early warning if a patient failed to attend 2 days in-a-row.

### Data collection and outcome measures

**Fig. 1:**
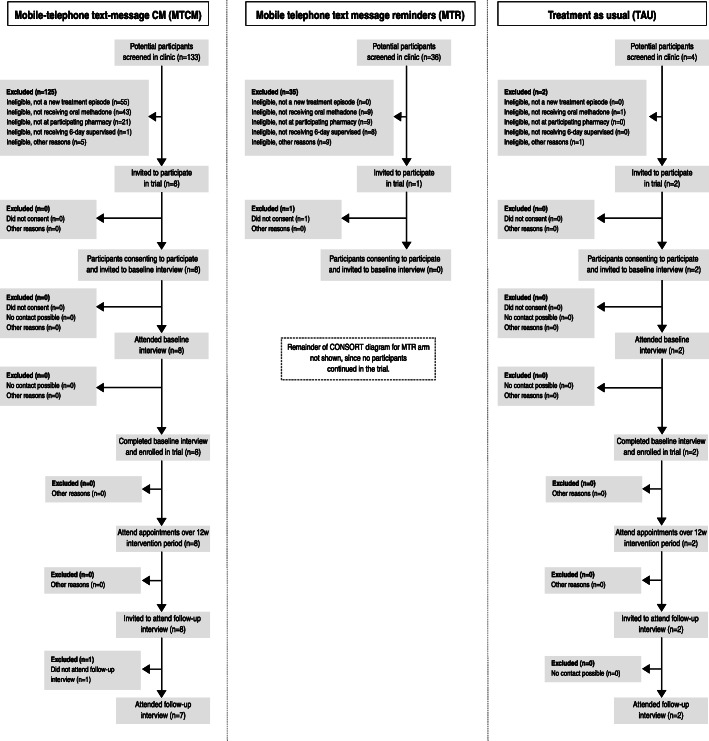
Consort diagram

There were four methods of data collection. First, the mobile telephone text message system recorded whether participants logged in each day via the tablet computer in the pharmacies. Second, pharmacy dispensing/supervision records—PharmOutcomes—a web-based system for capturing information on pharmacies services and financial tracking [16] relating to participants, were anonymised and provided to the research team. Third, a research interview with participants was undertaken by the research team at baseline (following consent) and again at 12–14 weeks post-enrolment (see Fig. 1). Fourth, as part of the process evaluation, focus groups were conducted between 10 and 12 weeks post-enrolment with participants and staff at two of the three drug services (delivering MTCM and TAU). Interviews were conducted at the end of the study with pharmacists at 6 of the 9 pharmacies. Participants received a £10 reimbursement for their time and travel at the baseline and follow-up interview, and at the focus group (if they attended).

### Feasibility outcomes

The primary feasibility outcome was the number of patients enrolled during each week of the 12-week intervention period, of those presenting for a new episode of OAT. Secondary feasibility outcomes included the following:

1. Number of drug services and pharmacies enrolled, relative to those approached.
2. Percentage of screened patients who were eligible and consenting to inclusion in the feasibility trial.
3. The accuracy of measuring pharmacy attendance, comparing participants’ self-login via tablet computer with the pharmacy record of daily supervised consumption using PharmOutcomes.
4. Adherence to telephone text message system, measured as the percentage of participants responding to a text messages sent weekly asking whether participants had received text messages in the previous week.
5. Rates of follow-up at 12–14 weeks.
6. Acceptability of the study to patients.

### Outcomes for use in a future confirmatory trial

Potential primary outcomes for use in a future confirmatory trial included the following:

7. Adherence to medication (using PharmOutcomes). The percentage of days adhering to medication by arm was measured as (i) percentage of days during 12-week intervention period when medication was taken, (ii) median number of days during 12-week intervention period when medication was not taken, (iii) Likert-like scale categorising participants by arm according to different missed dose patterns during 12 weeks post-enrolment, and (iv) time until a missed dose.
8. Aspects of the primary outcome measures needed for a sample size calculation for a future confirmatory trial including appropriate summary statistics (for example, mean and standard deviations for continuous outcomes), an estimate of the intraclass correlation (ICC) for the clusters, and qualitative information on the availability and usefulness of existing pharmacy dispensing data sets.

Secondary outcomes for use in a future confirmatory trial included the following:

9. Retention in treatment, measured as the number and percentage retained in treatment over the 12-week intervention period.
10. Illicit drug use measured by Opiate Treatment Index (Section 2—Drug Use) [17].
11. Alcohol Use Disorders Identification Test (AUDIT) [18].
12. Hospital Anxiety and Depression Scale (HADS) [19].
13. Social functioning measured using the Opiate Treatment Index (validated, mean social functioning subscale score) [20].
14. Physical and mental health status (short form-36 subscale mean scores) [21].
15. Sociodemographic characteristics, including age, gender, ethnicity, employment status, and living situation.
16. Outcomes for economic evaluation, including resource use schedules AD-SUS [22], EQ-5D-5L measure of health-related quality of life [23], and the ICECAP-A measure of capabilities [24].

### Process outcomes

Focus groups with participants and drug service staff and interviews with pharmacists were conducted to assess the acceptability of the intervention and trial procedures and to determine how contextual factors and treatment process impact on feasibility criteria (including recruitment, take-up, and compliance with assessments) the primary outcome for a future confirmatory trial (medication adherence).

#### Progression criteria to a future confirmatory trial included the following:

- Recruitment of three drug services, two to three pharmacies and 60 participants (20 from each drug service over 12 weeks).
- 50% of target patients (those patients presenting to participating drug services for a new episode of OAT who have not been receiving a prescription for methadone or other OAT medication for > 4 weeks and who have not been transferred in from another service or prison) eligible and consented.
- > 95% consistency in recording of pharmacy attendance (comparing daily pharmacy dispensing records vs. self-service internet login).
- Rates of follow-up at 12 weeks (> 70%).
- Completion rates of economic data collection (> 70%), missing (item missing/questionnaire missing) (< 10% missing data per questionnaire), and inconsistencies.

However, not achieving these criteria does not necessarily indicate unfeasibility of a future trial but might underline changes that need to be made to recruitment procedures, attendance record keeping, and resources for follow-up.

### Statistical and qualitative analysis

Feasibility outcomes were summarised using appropriate summary statistics (e.g. mean and standard deviation for normally distributed continuous outcomes; median and interquartile range for non-normal continuous outcomes; counts and proportions for categorical outcomes). The number and percentage of days when the two assessment methods (self-login via tablet computer vs. PharmOutcomes) were in agreement was calculated by (i) enumerating all days during each participant’s 12-week intervention period; (ii) removing days when the participant was paused, not supervised, or no longer receiving OST; and (iii) deriving a binary measure of agreement (matching = 1; not matching = 0). Appropriate summary statistics were calculated for secondary outcomes to be explored in a future confirmatory trial. Missing scale item data was handled as per specific recommendations for each questionnaire.

For the process evaluation, interviews and focus groups were based on a topic guide developed iteratively and applied flexibly to ensure coverage of key issues and responses to emergent themes. They were coded, transcribed, and analysed using thematic analysis in NVIVO.

## Results

### Feasibility outcomes

#### Percentage of drug services and pharmacies enrolled

Between July and December 2018, four drug services were approached (3 NHS, 1 non-NHS) and three (75%) were recruited (2 NHS, 1 non-NHS). Thirty-three allied community pharmacies were approached. Fifteen (46%) were excluded because they did not provide or rarely provided supervised consumption of oral methadone to patients at the drug clinic, and three (9%) did not respond to invitations to discuss the study. Of those eligible and responding (*n* = 15), nine (60%) were recruited and six (40%) declined to participate (for unknown reasons).

#### Percentage of screened patients who are eligible and consent to inclusion in the feasibility trial

One hundred and seventy-three patients starting a new treatment episode were screened for eligibility over a 12-week period between December 2018 and March 2019. The start of recruitment was delayed at the drug service assigned to MTR due to operational issues and recruited over a 6-week period only. The drug service had difficulties in identifying staff to conduct the screening and consent procedures. In total, 11 patients were eligible and 10 enrolled (6% of screened patients) (Fig. 1). One patient did not consent to participate in the feasibility trial. Numbers screened and enrolled varied across drug services: the MTCM arm screened 133 patients (unlike the other drug services, it screened those re-starting OAT after not receiving a prescription for < 4 weeks) and enrolled eight (6%); MTR arm screened 36 patients and enrolled no patients (0%); and in the TAU arm, four patients were screened and two enrolled (50%). The most frequent reasons for exclusion were (a) not a new treatment episode (MTCM group only) (34%; 55), (b) not receiving oral methadone (33%; 53), or (c) not attending a participating pharmacy (19%; 30). Six per cent were ineligible due to not receiving 6 days a week supervision (6%; 9), 4% not owning a mobile telephone (4%; 7), four failed to complete the screening/enrolment process (3%; 4), two were unable to read English (1%; 2), one was outside the catchment area (1), and one was unwilling to provide their mobile telephone number (1).

##### Sociodemographic characteristics

Participants were typically male (80%; 8), white (80%; 8), aged 41 years on average, unemployed/receiving sickness benefit (90%; 9), and living alone (40%; 4). They had all (100%) used heroin, crack, alcohol, and tobacco in the previous 30 days. Five (50%) were homeless. They had had similar characteristics to those receiving OAT (Table 1).

**Table 1:** Sociodemographic characteristics

Measure			MTCM (*n* = 8)	TAU (*n* = 2)	Total (*n* = 10)
Age		Mean (SD)	40.0 (9.6)	44.4 (18.7)	40.9 (10.7)
Gender	Female	*N* (%)	1 (12)	1 (50)	2 (20)
	Male	*N* (%)	7 (88)	1 (50)	8 (80)
Ethnicity	Caribbean	*N* (%)	1 (12)	0 (0)	1 (10)
	Other	*N* (%)	0 (0)	1 (50)	1 (10)
	Other White	*N* (%)	3 (38)	0 (0)	3 (30)
	White British	*N* (%)	4 (50)	1 (50)	5 (50)
Employment status	Employed	*N* (%)	0 (0)	1 (50)	1 (10)
	Unemployed/sickness benefit	*N* (%)	8 (100)	1 (50)	9 (90)
Living situation	Alone	*N* (%)	3 (38)	1 (50)	4 (40)
	Family	*N* (%)	1 (12)	0 (0)	1 (10)
	Other	*N* (%)	2 (25)	0 (0)	2 (20)
	Partner/spouse	*N* (%)	2 (25)	1 (50)	3 (30)

#### The accuracy of measuring pharmacy attendance

Table 2 presents the number and percentage of days when the two assessment methods were in agreement (i.e. either both indicating “attended/consumed” or both indicating “not attended/consumed”). Overall, between January and June 2019, 96% of days (517/538 days) were in agreement between both assessment methods (self-login vs. pharmacy records (PharmOutcomes)).

**Table 2:** Percentage of matches between attendance measured with pharmacy dispensing records (PharmOutcomes) vs. attendance recorded via self-login on tablet computer

	MTCM (*n* = 8)	TAU (*n* = 1)	Total (*n* = 9)
*N* (%) days where measures disagree	13 (2.7)	3 (6.0)	16 (3.0)
*N* (%) days where measures in agreement	472 (96.7)	45 (90.0)	517 (96.1)
*N* (%) days with missing data (on either measure)	3 (0.6)	2 (4.0)	5 (0.9)
Total days number of active days, all participants	488	50	538

#### Adherence to telephone text message system

Overall, most participants did not respond to the compliance text message (response rates were close to 0%). Due to concerns that poor response may have been due to the cost involved, an alternative message system was introduced after recruitment had ended that made it free for participants to reply, although responses improved only slightly. However, participants did contact the study team via the text message system regarding other issues. Towards the end of the study, this method of measuring compliance (i.e. relying on a response from participants) was supplemented with a more reliable method whereby the delivery status of all messages could be categorised retrospectively as “sent/not sent” and “delivered/not delivered”. Of 500 messages sent out over the entire study period, 383 (77%) were successfully delivered to participants. Reasons for unsuccessful delivery included losing mobile phone, mobile phone not charged, or mobile phone turned off to conserve charge.

#### Rates of follow-up at 12–14 weeks

Three participants stopped receiving the telephone system early in the study (due to entering inpatient detox (*n* = 1), moving to non-participating pharmacy (*n* = 1), or unsupervised take-home methadone (*n* = 1)). Overall, 9/10 (90%) participants attended follow-up interviews.

#### Acceptability to patients, staff, and pharmacists

This is reported under the “Process evaluation” section.

### Primary outcomes for use in a future confirmatory trial

#### Adherence to medication

Data were available for only 9/10 participants as one pharmacy was not able to provide PharmOutcomes data for their one participant. Participants in the MTCM arm adhered to their medication on 96% (464/485) of days and TAU on 58% (29/50) of days. The median number of missed days in the MTCM arm was 2 (IQR 4.5). In the TAU group, there was a single participant who missed 21 days in total during the intervention period. In each of the 12 weeks the telephone system was in use, most participants (across all arms) had no missed doses (Table 3). There was insufficient data to carry out repeated events survival analysis of time until missed dose.

**Table 3:** Categories of missed response patterns, for each week of intervention period, all arms combined (*n* = 9)

Week of intervention	No missed doses	Missed 1 or 2 doses	Missed 3+ doses	Total active participants this week
	*N* (%)	*N* (%)	*N* (%)	*N*
1	8 (89)	1 (11)	0 (0)	9
2	6 (67)	3 (33)	0 (0)	9
3	6 (75)	2 (25)	0 (0)	8
4	5 (71)	1 (14)	1 (14)	7
5	4 (57)	2 (29)	1 (14)	7
6	4 (57)	3 (43)	0 (0)	7
7	4 (57)	2 (29)	1 (14)	7
8	5 (83)	1 (17)	0 (0)	6
9	5 (100)	0 (0)	0 (0)	5
10	4 (67)	1 (17)	1 (17)	6
11	4 (80)	1 (20)	0 (0)	5
12	5 (100)	0 (0)	0 (0)	5

#### Aspects of primary outcomes needed for a sample size calculation

Given the small numbers of participants, and only two clusters with enrolled participants, it was not possible to calculate the intraclass correlation for clusters.

#### Usefulness of data sets

Extracts of data from the telephone system were automated. In contrast, PharmOutcomes data was provided by the pharmacies in various formats, making it necessary for these data to be re-entered into the databases by hand.

### Secondary outcomes for use in a future confirmatory trial

#### The number and percentage of participants retained in treatment

The number and percentage of participants retained in treatment (i.e. remaining at the same clinic and still receiving oral methadone) over the 12-week intervention period relative to those enrolled can be seen in Table 4. Nine out of 10 continued to receive treatment at the same clinic, and 2/10 received a different OST. One participant did not attend their follow-up interview.

**Table 4:** Retention in treatment at follow-up, by arm

		*N* (%)		
		MTCM	TAU	Total
Retained in treatment	Supervised same clinic	6 (75)	1 (50)	7 (70)
	Different OST same clinic	1 (12)	1 (50)	2 (20)
Unknown	Did not attend follow-up interview	1 (12)	0 (0)	1 (10)
Total		8	2	10

#### Illicit drug use and clinical outcomes

Illicit drugs used and clinical outcomes in past 30 days are presented in the supplementary material. The MTR group are not presented in these tables because no participants were enrolled to this arm. No items were missing in any of the questionnaires.

#### Health economics

Four items were missing from the baseline AD-SUS health economics questionnaire addressing service contact; one item at follow-up across all participants. While we recognise that some feasibility studies do take the opportunity to cost the intervention, we prefer instead to do this as part of a subsequent full randomised controlled trial (RCT) in order to provide the most up to date evidence of intervention cost, at a point after the intervention has been through all processes of testing, modification, and testing. Doing this at the feasibility stage risks estimating the cost of an intervention that may be adapted or changed as part of the feasibility trial, and thus, the costs may not reflect the reality of the intervention when tested in a later stage RCT.

##### Adverse events

There were nineteen adverse events among six participants (4× MTCM; 2× TAU), one of which was a serious adverse event (1× MTCM). None were related to the intervention.

### Process evaluation

#### Focus groups and interviews with participants, staff, and pharmacists

In focus groups conducted with participants in the MTCM arm, participants were positive about the telephone system. Participants welcomed the praise messages (one message per day) and found them encouraging, helpful, constructive, and supportive. Participants found the tablet device easy and straightforward to use and noted that it encouraged greater and more positive interaction with their pharmacist. The use of debit cards was well received, and participants highlighted the importance of achieving the weekly bonus payment. Staff prescribers in the MTI group reported that the telephone system had a positive impact on their service. The weekly emails they received allowed for close monitoring of their patients, maintaining a rapport with them and saving staff time not having to call pharmacies. The email alerts enabled them to know immediately of missed doses. Pharmacists reported that the tablet device was quick and easy to use, it became part of a routine, and did not impact on the pharmacy. In interviews conducted with staff in all three arms, staff acknowledged the low numbers of new opiate users presenting to services. In particular, staff in the TAU group considered the very low numbers of opioid users presenting to the drug service during the recruitment period to be “highly unusual” for their drug service. Staff suggested changes to eligibility criteria in a future confirmatory trial, specifically to include existing OAT patients repeatedly missing doses as well as patients prescribed buprenorphine.

#### Issues with the telephone system or debit card

There were only a few instances of participants being unable to self-login at the pharmacy, and these were quickly resolved early on in the study. Two participants lost their mobile telephones over 82 days and were unable to receive text messages, and two participants lost their debit cards which were easily replaced.

## Discussion

CM delivered by mobile telephone to encourage medication adherence with OAT was feasible to deliver, well-liked by patients and drug service staff, and acceptable to pharmacists. There was good consistency in recording of pharmacy attendance and dose consumption between patient self-login and pharmacy prescribing records. Weekly reports of attendance and early warning of missed doses were also well-liked by patients and drug service staff who reported a positive impact on patient care. While it was feasible to recruit drug services and pharmacists, it was not feasible to recruit the target number of 60 individuals entering OAT. Good rates of follow-up and data completeness were achieved.

With the huge increase in mobile telephone use and coverage, there has been interest in developing mobile health interventions in general, including CM [25]. CM has previously been adapted to be delivered remotely with the aim of providing treatment to patients who are not attending treatment services or attending intermittently to support recovery. A recent systematic review and meta-analysis of contingency management delivered by mobile telephone concluded CM delivered by mobile telephones is effective in reducing substance use among patients with tobacco and alcohol use disorder [13]. There has been one previous study which found mobile telephone delivered CM improved HIV medication adherence among individuals with substance use disorder and HIV [26]. This is the first study of mobile telephone CM in the UK and the first targeted at OAT patient’s adherence to medication.

Patient self-login at community pharmacies was acceptable and well-liked by patients, staff, and pharmacists. Patients found the tablet device easy and straightforward to use and noted that it encouraged greater and more positive interaction with their pharmacist. Regarding daily recording attendance and consumption of medication, there was a 96% consistency between patient self-logins and pharmacy dispending records. However, this result should not be over-interpreted given the small sample size. As we only have data from MTCM and very few from TAU, we do not know if participants not receiving incentives will login to tablet devices. In particular, patients liked the reminders, the message content, and the 6-day bonus system. Participants welcomed the praise messages and found them encouraging, helpful, constructive, and supportive.

Extracting data from the telephone system was automated and would scale-up well. In contrast, PharmOutcomes data (or equivalent) will need to be obtained on a larger scale and in a consistent format that can easily be imported into trial databases (i.e. avoiding time-consuming manual cleaning and re-entry).

The automatic weekly reports of attendance and early warning of missed doses provided to prescribers were an innovative and useful clinical addition to mobile telephone CM. They were well-liked by both patients and staff. Allowing staff to closely monitor their patients, maintain a rapport with them, and save them time normally spent calling pharmacies for updates on patient attendance and missed doses. The early warning alerts provided prescribers with an immediate report of missed doses. Pharmacists are not usually asked to alert prescribers when a patient has missed one or two doses, so this function has obvious benefits in helping prescribers to provide informed and responsive treatment. Patients valued having evidence of their medication adherence.

While the study found that drug services and pharmacies could successfully be recruited to a cluster trial, only 10 of the target 60 participants were recruited. The study was unable to recruit the target patient population due to lower than expected numbers of patients presenting for OAT, higher than expected numbers receiving buprenorphine, and higher numbers than expected of allied pharmacies, at the time of designing the study. In interviews, staff at all services acknowledged the low numbers of new opioid users presenting to drug services, although the very low numbers in TAU were considered highly unusual. Moreover, screening at the MTR clinic was undertaken over 6 weeks instead of the planned 12 due to unexpected recruitment issues and thus limiting numbers of potential participants.

Recruitment could be improved in a future confirmatory trial by revising the study patient eligibility criteria to target existing OAT patients who are repeatedly missing doses of methadone and buprenorphine and increase the number of participating pharmacies for each drug clinic site. Staff considered these to be important patient groups to target. We plan to undertake some additional work in preparation for a future main trial. This will include identifying the numbers (and characteristics) of existing OAT (methadone and buprenorphine) patients who miss 3 or more days doses at drug clinics in England to ensure we are able to recruit our revised target population. We will use these data to conduct a sample size calculation for inform a future trial.

When planning a future confirmatory trial, we propose to (a) target a different population of existing clients in OAT, including patients who have missed their OAT doses for > 3 days or are receiving buprenorphine as well as methadone; (b) increase the number of participating pharmacies allied to each drug service; and (c) identify alternative ways of accessing PharmOutcomes data/supervised medication records and (d) ensure participants not receiving incentives (in the reminder and TAU groups) login to tablet devices and there is a high degree of accuracy with pharmacy dispensing and supervision records (Table 5). These elements could be assessed with an internal pilot. An internal pilot can also help to identify unexpected recruitment issues.

**Table 5:** Progression criteria to a future confirmatory trial

	Progression criteria	Achieved yes/no
1.	Recruitment of 3 drug services	Yes
2.	Recruitment of 2–3 pharmacies per clinic	Yes
3.	60 participants enrolled (20 from each clinic over 12 weeks)	No, only 10 participants enrolled
4.	50% target patients (presenting to participating drug services for a new episode of OST) eligible and consented	No, only 9% of target population were eligible and consented due to low numbers presenting for OST at all clinics and high numbers excluded due to receiving buprenorphine and not attending a participating pharmacy
5.	> 95% consistency in recording of pharmacy attendance (comparing daily pharmacy dispensing records vs. patient self-login)	Yes, achieved 96%
6.	Rates of follow-up at 12 weeks (> 70%)	Yes, achieved 90% (9/10)
7.	Completion rates of economic data collection (> 70%)	Yes, achieved 100%
8.	Missing (< 10% missing data per questionnaire)	Yes

## Conclusions

Overall, while the intervention proved to be feasible to implement and deliver and was well-liked by drug service staff, pharmacists, and patients, recruitment rates sufficient for a definitive trial were not feasible and remain a challenge to be addressed. Mobile telephone CM to improve adherence to medication has shown promise, and using technology to deliver interventions is at the core of improving healthcare in NHS England’s Five Year Forward View. We propose that a confirmatory trial to assess the clinical and cost-effectiveness is now needed.

## Supplementary Information

**Additional file 1.** Supplementary material.

## Data Availability

Data sharing: Data collected for the study, including individual participant data and a data dictionary defining each field in the set, will be made available to others. These data will include deidentified participant data, data dictionary, study protocol, statistical analysis plan, and informed consent form. These data will be available with publication and on application to and approval from Dr Nicola Metrebian with study proposal and with a signed data access agreement.
