# Supplementary material for: Mobile telephone delivered contingency management for encouraging adherence to supervised methadone consumption: feasibility study for an RCT of clinical and cost-effectiveness (TIES)
Source: Pilot Feasibility Stud. 2021 Jan 7;7:14. doi: 10.1186/s40814-020-00761-4 (PMC7789356; doi:10.1186/s40814-020-00761-4)
Supplement: Supplementary file 1 — Additional file 1. Supplementary material. [file 40814_2020_761_MOESM1_ESM.docx]

**Supplementary material**

**Table i. Use of illicit drugs at baseline and follow-up**

|  | **Use of illicit street drugs, past 30 days** | | | | | | | | **Number of days used, past 30 days** | | | | | | | |
| --- | --- | --- | --- | --- | --- | --- | --- | --- | --- | --- | --- | --- | --- | --- | --- | --- |
|  | N complete / N total | | | | N using, last 30  days / N complete | | | | N complete / N total | | | | Median (IQR) days used,  last 30 days | | | |
|  | CM | TAU | R | Total | CM | TAU | R | Total | CM | TAU | R | Total | CM | TAU | R | Total |
| Alcohol | 8/8 (100) | 2/2 (100) | - (-) | 10/10 (100) | 4/8 (50) | 1/2 (50) | - (-) | 5/10 (50) | 8/8 (100) | 2/2 (100) | -- | 10/10 (100) | 2 (17.0) | 15 (15.0) | - (-) | 2 (19.0) |
| Amphetamines | 8/8 (100) | 2/2 (100) | - (-) | 10/10 (100) | 0/8 (0) | 0/2 (0) | - (-) | 0/10 (0) | 8/8 (100) | 2/2 (100) | -- | 10/10 (100) | - (-) | - (-) | - (-) | - (-) |
| Benzodiazepines | 8/8 (100) | 2/2 (100) | - (-) | 10/10 (100) | 0/8 (0) | 0/2 (0) | - (-) | 0/10 (0) | 8/8 (100) | 2/2 (100) | -- | 10/10 (100) | - (-) | - (-) | - (-) | - (-) |
| Cannabis | 8/8 (100) | 2/2 (100) | - (-) | 10/10 (100) | 6/8 (75) | 1/2 (50) | - (-) | 7/10 (70) | 8/8 (100) | 2/2 (100) | -- | 10/10 (100) | 22 (18.0) | 15 (15.0) | - (-) | 22 (26.0) |
| Cocaine | 8/8 (100) | 2/2 (100) | - (-) | 10/10 (100) | 1/8 (12) | 1/2 (50) | - (-) | 2/10 (20) | 8/8 (100) | 2/2 (100) | -- | 10/10 (100) | 0 (0.0) | 8 (7.5) | - (-) | 0 (0.0) |
| Codeine | 8/8 (100) | 2/2 (100) | - (-) | 10/10 (100) | 1/8 (12) | 1/2 (50) | - (-) | 2/10 (20) | 8/8 (100) | 2/2 (100) | -- | 10/10 (100) | 0 (0.0) | 15 (15.0) | - (-) | 0 (0.0) |
| Crack | 8/8 (100) | 2/2 (100) | - (-) | 10/10 (100) | 7/8 (88) | 2/2 (100) | - (-) | 9/10 (90) | 8/8 (100) | 2/2 (100) | -- | 10/10 (100) | 30 (0.0) | 30 (0.5) | - (-) | 30 (0.0) |
| Diamorphine | 8/8 (100) | 2/2 (100) | - (-) | 10/10 (100) | 0/8 (0) | 0/2 (0) | - (-) | 0/10 (0) | 8/8 (100) | 2/2 (100) | -- | 10/10 (100) | - (-) | - (-) | - (-) | - (-) |
| Ecstasy/MDMA | 8/8 (100) | 2/2 (100) | - (-) | 10/10 (100) | 0/8 (0) | 1/2 (50) | - (-) | 1/10 (10) | 8/8 (100) | 2/2 (100) | -- | 10/10 (100) | 0 (0.0) | 0 (0.5) | - (-) | 0 (0.0) |
| Heroin | 8/8 (100) | 2/2 (100) | - (-) | 10/10 (100) | 8/8 (100) | 2/2 (100) | - (-) | 10/10 (100) | 8/8 (100) | 2/2 (100) | -- | 10/10 (100) | 30 (0.0) | 26 (4.5) | - (-) | 30 (0.0) |
| Injectable Methadone | 8/8 (100) | 2/2 (100) | - (-) | 10/10 (100) | 0/8 (0) | 0/2 (0) | - (-) | 0/10 (0) | 8/8 (100) | 2/2 (100) | -- | 10/10 (100) | - (-) | - (-) | - (-) | - (-) |
| Legal Highs | 8/8 (100) | 2/2 (100) | - (-) | 10/10 (100) | 0/8 (0) | 0/2 (0) | - (-) | 0/10 (0) | 8/8 (100) | 2/2 (100) | -- | 10/10 (100) | - (-) | - (-) | - (-) | - (-) |
| Mephedrone | 8/8 (100) | 2/2 (100) | - (-) | 10/10 (100) | 0/8 (0) | 0/2 (0) | - (-) | 0/10 (0) | 8/8 (100) | 2/2 (100) | -- | 10/10 (100) | - (-) | - (-) | - (-) | - (-) |
| Morphine | 8/8 (100) | 2/2 (100) | - (-) | 10/10 (100) | 0/8 (0) | 0/2 (0) | - (-) | 0/10 (0) | 8/8 (100) | 2/2 (100) | -- | 10/10 (100) | - (-) | - (-) | - (-) | - (-) |
| Oral Methadone | 8/8 (100) | 2/2 (100) | - (-) | 10/10 (100) | 4/8 (50) | 0/2 (0) | - (-) | 4/10 (40) | 8/8 (100) | 2/2 (100) | -- | 10/10 (100) | 0 (1.2) | 0 (0.0) | - (-) | 0 (1.0) |
| Tobacco | 8/8 (100) | 2/2 (100) | - (-) | 10/10 (100) | 8/8 (100) | 2/2 (100) | - (-) | 10/10 (100) | 6/8 (75) | 2/2 (100) | -- | 8/10 (80) | 30 (0.0) | 30 (0.0) | - (-) | 30 (0.0) |

**Table ii:** *Mean (SD) for AUDIT scale at baseline and follow-up*

| Trial arm |  | N complete / N total | Mean (SD) |
| --- | --- | --- | --- |
| MTCM | Baseline (0w) | 8/8 | 5.1 (7.5) |
|  | Follow-up (12w) | 7/7 | 4.4 (5.0) |
| TAU | Baseline (0w) | 2/2 | 15.5 (21.9) |
|  | Follow-up (12w) | 2/2 | 6.5 (6.4) |

**Table iii** HADS total anxiety and depression subscale scores, by arm

|  | | **Anxiety subscale** | | **Depression subscale** | |
| --- | --- | --- | --- | --- | --- |
| Trial arm | | N complete /  N total | Mean (SD) | N complete /  N total | Mean (SD) |
| MTCM | Baseline (0w) | 8/8 | 12.8 (3.6) | 8/8 | 11.5 (3.5) |
|  | Follow-up (12w) | 7/7 | 10.4 (4.8) | 7/7 | 9.9 (3.3) |
| TAU | Baseline (0w) | 2/2 | 9.5 (10.6) | 2/2 | 6.5 (7.8) |
|  | Follow-up (12w) | 2/2 | 16.5 (0.7) | 2/2 | 10.5 (0.7) |

**Table iv.** OTI social functioning subscale, by arm

| Trial arm |  | N complete / N total | Mean (SD) |
| --- | --- | --- | --- |
| MTCM | Baseline (0w) | 8/8 | 26.1 (3.0) |
|  | Follow-up (12w) | 7/7 | 22.1 (3.8) |
| TAU | Baseline (0w) | 2/2 | 20.5 (7.8) |
|  | Follow-up (12w) | 2/2 | 25.0 (11.3) |

**Table v.** *SF-36v2 mental and physical component summary scores*

|  | | Mental component summary score (MCS) | | Physical component summary score (PCS) | |
| --- | --- | --- | --- | --- | --- |
| Trial arm |  | N complete /  N attending interview | Mean (SD) | N complete /  N attending interview | Mean (SD) |
| MTCM | Baseline (0w) | 8/8 | 30.5 (11.0) | 8/8 | 48.8 (10.4) |
|  | Follow-up (12w) | 7/7 | 32.0 (12.6) | 7/7 | 54.7 (9.5) |
| TAU | Baseline (0w) | 2/2 | 35.2 (23.2) | 2/2 | 40.3 (2.3) |
|  | Follow-up (12w) | 2/2 | 15.2 (8.0) | 2/2 | 44.2 (12.5) |
